# Efflux Pump, the Masked Side of ß-Lactam Resistance in *Klebsiella pneumoniae* Clinical Isolates

**DOI:** 10.1371/journal.pone.0004817

**Published:** 2009-03-12

**Authors:** Jean-Marie Pages, Jean-Philippe Lavigne, Véronique Leflon-Guibout, Estelle Marcon, Frédéric Bert, Latifa Noussair, Marie-Hélène Nicolas-Chanoine

**Affiliations:** 1 UMR-MD-1, Facultés de Médecine et de Pharmacie, Université de la Méditerranée, IFR88, Marseille, France; 2 AP-HP, Hôpital Beaujon, Service de Microbiologie, Clichy, France; 3 INSERM U 773, Centre de Recherche Biomédicale Bichat-Beaujon, CRB3, Université D. Diderot, Paris, France; Baylor College of Medicine, United States of America

## Abstract

**Background:**

β-lactamase production and porin decrease are the well-recognized mechanisms of acquired ß-lactam resistance in *Klebsiella pneumoniae* isolates. However, such mechanisms proved to be absent in *K. pneumoniae* isolates that are non susceptible to cefoxitin (FOX) and succeptible to amoxicillin+clavulanic acid in our hospital. Assessing the role of efflux pumps in this β-lactam phenotype was the aim of this study.

**Methodology/Findings:**

MICs of 9 β-lactams, including cloxacillin (CLX), and other antibiotic families were tested alone and with an efflux pump inhibitor (EPI), then with both CLX (subinhibitory concentrations) and EPI against 11 unique bacteremia *K. pneumoniae* isolates displaying the unusual phenotype, and 2 ATCC strains. CLX and EPI-dose dependent effects were studied on 4 representatives strains. CLX MICs significantly decreased when tested with EPI. A similar phenomenon was observed with piperacillin+tazobactam whereas MICs of the other β-lactams significantly decreased only in the presence of both EPI and CLX. Thus, FOX MICs decreased 128 fold in the *K. pneumoniae* isolates but also16 fold in ATCC strain. Restoration of FOX activity was CLX dose-dependent suggesting a competitive relationship between CLX and the other β-lactams with regard to their efflux. For chloramphenicol, erythromycin and nalidixic acid whose resistance was also due to efflux, adding CLX to EPI did not increase their activity suggesting differences between the efflux process of these molecules and that of β-lactams.

**Conclusion:**

This is the first study demonstrating that efflux mechanism plays a key role in the β-lactam susceptibility of clinical isolates of *K. pneumoniae*. Such data clearly evidence that the involvement of efflux pumps in ß-lactam resistance is specially underestimated in clinical isolates.

## Introduction

*Klebsiella pneumoniae*, a member of *Enterobacteriaceae* family, is an important pathogen in both the community and the hospital setting [1]. Over the last 20 years, attention has particularly been paid to *K. pneumoniae* K1 isolates causing pyogenic liver abscesses and also to multidrug-resistant *K. pneumoniae* isolates, primarily observed in the hospital and currently also in the community [1]–[3].

β-lactamase production and reduced porin levels are the main mechanisms of ß-lactam resistance reported in *K. pneumoniae* isolates [4]. We recently observed clinical isolates with a paradoxal β-lactam phenotype suggesting mechanisms not linked to β-lactamase acquisition: these isolates were less susceptible to cefoxitin and susceptible to both amoxicillin/clavulanic acid and extended-spectrum cephalosporins. Cefoxitin resistance in enterobacterial isolates without chromosomal AmpC β-lactamase suggests the production of an AmpC-plasmid-mediated enzyme. This mechanism could not be retained because the isolates were susceptible to amoxicillin/clavulanic acid which is inconsistent with a plasmid-mediated-AmpC production [5]. Alternatively, the β-lactam-resistant phenotype and the chloramphenicol and nalidixic acid resistance may suggest a modification of the membrane permeability in the isolates [6]. The final mechanism which may be involved in cefoxitin resistance is active efflux. Such a possibility has recently been hypothesized, but not yet demonstrated [7].

An analysis of the microbiology hospital laboratory database showed that this unusual β-lactam phenotype was present in almost 5% of all *K. pneumoniae* clinical isolates in our hospital for the last 8 years. These isolates were epidemiologically unrelated, obtained from various specimens (urine 41%, deep-seated pus and fluids 20%, blood 14%, drainage fluids 8%, broncho-pulmonary aspirates 4%, other 13%) and originated from different hospital wards (surgery 33%, medicine 31%, intensive care units (ICU) 25%, emergency 9%, and obstetrics 2%).

The main purpose of this study was to assess the role of efflux pumps in the unusual β-lactam resistant phenotype that is emerging in clinical isolates.

## Results

### Molecular typing and β-lactamase content of isolates

A unique RAPD profile was found from each *K. pneumoniae* isolate (Table 1). No TEM- and AmpC- encoding genes were detected in these isolates.

**Table 1:** Clinical and molecular characters of 11 *K. pneumoniae* clinical isolates less susceptible to cefoxitin and susceptible to amoxicillin+clavulanic acid.

Strain	Sample	Isolation date	Ward	RAPD profile	Chromosomal *bla* gene
KPBj 1	Blood	07/31/2003	Hematology	G	SHV-1
KPBj 2	Blood	06/05/2004	Digestive surgery	D	SHV-1
KPBj 3	Blood	11/15/2001	Hepatology	F	LEN-1
KPBj 4	Blood	11/30/2000	Hepatology ICU	C	SHV-1
KPBj 5	Blood	06/15/2004	Surgery ICU	H	SHV-11
KPBj 6	Drainage Fluid	05/29/2005	Hepatology	J	SHV-1
KPBj 7	Blood	02/03/2005	Hepatology ICU	I	SHV-1
KPBj 8	Blood	10/08/2001	Digestive ICU	E	SHV-1
KPBj 9	Blood	08/27/2001	Digestive ICU	K	SHV-1
KPBj 10	Blood	06/21/2000	Hepatology ICU	A	LEN-1
KPBj 11	Blood	08/23/2000	Gastro-enterology	B	OKP-B

ICU: intensive care unit.

The 3 chromosomal *bla* gene types previously described in *K. pneumoniae* [8] were found in all isolates, the most common type (n = 8) being *bla*_SHV_ (Table 1).

### Immunodetection of AcrA-TolC components and porins

**Figure 1:**
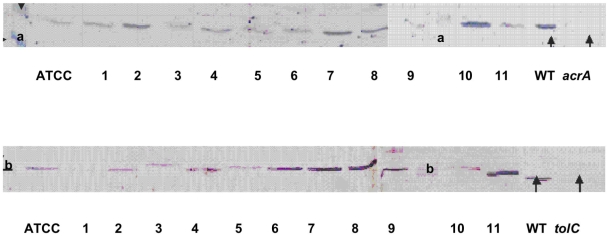
Detection of AcrA and TolC in *Klebsiella pneumoniae* isolates. Top part, immunodetection was carried out with antiserum directed against denatured AcrA; bottom part, immunodetection was carried out with antiserum directed against denatured TolC porin. WT, *E. aerogenes* wild type strain producing normal level of AcrA and TolC; *acrA*, *acrA* deleted strain; *tolC*, *tolC* deleted strain [16]. a and b indicate the migration of the molecular weight marker 30 kD and 43 kD, respectively. ATCC, ATCC11296, lanes 1 to 11, strain KPBj 1 to strain KPBj 11.

To investigate a possible alteration of membrane permeability due to an increase in the major RND efflux pump, AcrAB [6], [9], [10], and a decrease in porin production [11], immunodetection analyses were performed on clinical isoates. Concerning the efflux mechanism, five (KPBj 1, 3, 5, 9, and 11) of the eleven isolates expressed AcrA at a level similar to that of the reference strain ATCC 11296 and six (KPBj 2, 4, 6, 7, 8, and 10) at a higher level (Figure 1). Regarding TolC, four strains (KPBj 6, 7, 8, and 11) exhibited a higher signal than that of strain ATCC 11296 (Figure 1).

**Figure 2:**
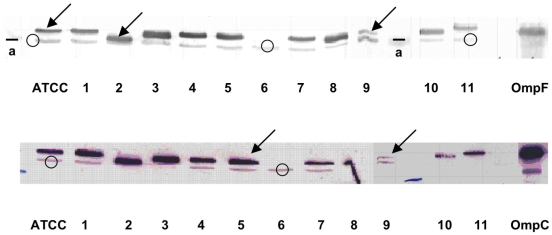
Detection of porins in *Klebsiella pneumoniae* isolates. Top part, immunodetection was carried out with antiserum directed against denatured OmpF porin; bottom part, immunodetection was carried out with antiserum directed against denatured OmpC porin. In the two incubation assays, antiserum directed against denatured OmpA was used as control. ATCC: ATCC11296; lane 1 to 11, strain KPBj 1 to strain KPBj 11; OmpF and OmpC illustrate the migration of the *E. coli* porins in the same conditions (used as internal standards in SDS-PAGE). Arrows and circles indicate the migration of porins and OmpA, respectively; a, indicates the migration of the molecular weight marker (30 kD).

For porin expression, all isolates except for strain KPBj 6, exhibited a positive signal with variable intensity when the detection was carried out with the antisera directed against porins (Figure 2). Similar results were obtained with a specific antibody directed against the internal loop of enterobacterial porins (data not shown). In contrast, OmpA was detected in all isolates (Figure 2).

### Antibiotic susceptibility

The *K. pneumoniae* isolates showed an extended multidrug-resistance profile according to MICs of the antibiotics tested (Table 2). Most isolates exhibited a resistance level to chloramphenicol, nalidixic acid, amoxicillin, piperacillin and cefoxitin. MICs of cloxacillin and erythromycin, two antibiotics known to be non active on *Enterobacteriaceae*, were extremely high. The absence of mutations in the quinolone resistance determining region (QRDR) of GyrA and ParC as well as the absence of *qnr* genes was checked in the eleven isolates (data not shown).

**Table 2:** MICs of various antibiotics tested alone and with efflux inhibitor PAβN* towards 11 *K. pneumoniae* clinical isolates and ATCC strains.

Strain	CMI mg/L																									
	CMP		NAL		OFX		AMX		AMC		PIP		TZP		FOX		CAZ		FEP		ERT		CLX		ERY	
	−	+	−	+	−	+	−	+	−	+	−	+	−	+	−	+	−	+	−	+	−	+	−	+	−	+
KPBj 1	4	2	16	1	0.125	0.03	2048	2048	4	4	16	16	4	1	8	8	0.25	0.25	0.25	0.25	0.03	0.03	1024	64	64	1
KPBj 2	64	4	32	1	0.5	0.06	512	512	8	8	64	64	8	4	64	32	1	0.5	1	1	0.015	0.015	2048	64	512	1
KPBj 3	32	4	64	1	0.25	0.03	2048	2048	2	2	4	4	4	1	16	16	0.25	0.25	0.5	0.5	0.015	0.015	512	64	128	2
KPBj 4	512	8	512	1	2	0.03	2048	2048	2	2	16	16	8	2	64	32	0.5	0.25	0.5	0.5	0.015	0.03	1024	64	128	1
KPBj 5	256	4	64	1	1	0.06	512	512	4	4	32	32	4	2	16	8	0.5	0.25	1	1	0.015	0.03	1024	128	256	1
KPBj 6	512	4	2048	4	16	0.5	1024	1024	4	4	64	64	8	1	64	64	0.5	0.5	0.5	0.5	0.25	0.25	2048	128	128	1
KPBj 7	128	8	128	4	64	0.5	512	512	8	8	64	64	16	4	64	64	1	0.5	0.5	0.5	0.06	0.06	2048	256	512	2
KPBj 8	32	4	64	2	0.5	0.06	1024	1024	8	8	32	32	8	4	64	64	1	0.5	1	1	0.5	0.125	2048	128	128	1
KPBj 9	16	2	64	4	0.5	0.125	2048	2048	4	4	128	128	128	64	128	64	1	0.5	1	0.5	0.25	0.25	1024	64	128	0.25
KPBj 10	64	4	64	2	1	0.25	512	512	8	8	32	16	8	4	128	64	1	0.5	1	1	0.015	0.015	2048	256	512	4
KPBj 11	128	4	128	2	2	0.25	1024	1024	4	4	64	32	4	2	64	32	0.5	0.5	0.25	0.25	0.03	0.015	2048	128	512	2
ATCC 11296	8	2	16	1	0.25	<0.01	256	128	2	2	32	32	4	1	8	8	1	0.25	0.06	0.06	0.015	0.015	1024	64	128	0.25
ATCC 138821	nd	nd	nd	nd	nd	nd	256	256	4	4	16	16	nd	nd	8	8	1	0.25	0.125	0.125	0.015	0.015	1024	64	nd	nd

*: 0.096 mM (50 mg/L), CMP: chloramphenicol, NAL: nalidixic acid, OFX: ofloxacin, AMX: amoxicillin, AMC: amoxicillin+clavulanic acid, PIP: piperacillin, TZP: piperacillin+tazobactam, FOX: cefoxitin, CAZ: ceftazidime, FEP: cefepime, ERT: ertapenem, CLX: cloxacillin, ERY: erythromicin, +: with PAβN, −: without PAβN, nd: not determined.

We have previously demonstrated that phenylalanine-arginine ß-naphthylamide (PAßN) is able to block the efflux pumps involved in antibiotic expel in *E. aerogenes* and *Klebsiella pneumoniae* clinical strains [12], [13]. This efflux pump inhibitor is able to restore partially or totally, depending on the presence of additional resistance mechanisms, the susceptibility to several antibiotics in resistant isolates and it can be fruitfully used to detect the presence of an inhibitor-sensitive efflux in resistant enterobacterial strains [14]–[16]. Adding the efflux inhibitor PAßN resulted in a significant decrease in MICs of some antibiotics to which wild type *K. pneumoniae* isolates are susceptible - chloramphenicol, nalidixic acid, ofloxacin - and also to which *K. pneumoniae* isolates are inherently resistant - cloxacillin and erythromycin (Table 2). On the contrary, the addition of PAßN did not significantly modify MICs of the ß-lactam molecules tested - amoxicillin alone and associated with clavulanic acid (an inhibitor of class A β-lactamases), piperacillin, cefoxitin, ceftazidime, cefepime and ertapenem, - except for piperacillin associated with tazobactam, another inhibitor of class A β-lactamases (Table 2).

### ß-lactam susceptibility and competitive assays

With the same strains and under the same conditions, we observed a 4 to 128-fold decrease in cefoxitin MICs tested in the presence of PAßN when cloxacillin was added at sub-inhibitory concentrations (1/20 MIC). Such an addition led to no variation of nalidixic acid MICs tested in the presence of PAßN (Table 3). The reduction of the MIC for cefoxitin by PAßN was less effective in strain KPBj 6 which was porin deficient (Table 3).

**Table 3:** MICs of cefoxitin and nalidixic acid tested alone and with inhibitor efflux (PAβN) and sub-inhibitory concentrations of cloxacillin towards 4 representative *K. pneumoniae* strains.

Strain	MIC mg/L									
	FOX	NAL	CLX	FOX+	NAL+	CLX+	FOX+	NAL+	FOX+	NAL+
				PAβN*	PAβN*	PAβN*	CLX^♦^	CLX^♦^	CLX^♦^+PAβN*	CLX^♦^+PAβN*
ATCC 11296	8	16	1024	8	1	64	4	16	0.25	1
KPBj 6	64	2048	2048	64	4	128	32	1024	8	8
KPBj 7	64	128	2048	64	4	256	64/32	64	0.5	4
KPBj 9	128	64	1024	64	4	64	64	128	1	4

FOX: cefoxitin, NAL: nalidixic acid, CLX: cloxacillin, *: 0.096 mM (50 mg/L), ^♦^: 1/20 CLX MIC.

**Figure 3:**
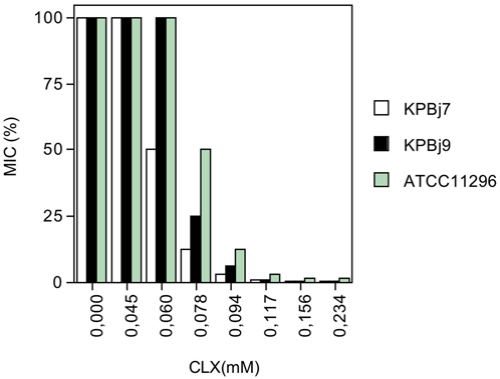
MICs of cefoxitin for two *K. pneumoniae* isolates (KpBj 7 and 9) and strain ATCC 11296 measured in the presence of efflux inhibitor PAβN (0.096 mM) and increased concentrations of cloxacillin (CLX). MIC percent (MIC%) represents the decrease in cefoxitin MIC in the presence of different CLX concentrations (plotted as CLX (mM)) compared with MIC measured without PAßN and CLX.

To characterize the apparent competitive efflux between cefoxitin and cloxacillin in the presence of PAßN, a cloxacillin dose-effect experience was carried out (Figure 3). At a cloxacillin concentration of 0.094 mM, cefoxitin MICs were significantly reduced against all strains. In contrast, no variation was detected when the competition assay was carried out with nalidixic acid (Table 4).

**Table 4:** Cloxacillin dose-effect on MICs of nalidixic acid tested alone and with PAβN towards 4 representative *K. pneumoniae* strains.

Strain	MIC (mg/L) of NAL								
	Alone	+PAßN*	+PAβN*+CLX [mM]						
			0.045	0.06	0.078	0.096	0.117	0.156	0.234
ATCC 11296	16	1	1	1	1	1	1	1	1
KPBj 6	2048	4	4	4	4	4	8	8	8
KPBj 7	128	4	4	4	4	4	4	4	4
KPBj 9	64	4	4	4	4	4	4	4	4

NAL: nalidixic acid, *: 0.096 mM (50 mg/L), CLX: cloxacillin.

To determine a possible concentration threshold for PAßN effect on cefoxitin MIC in the presence of cloxacillin, a dose assay was carried out with increasing PAßN concentrations and two different cloxacillin concentrations. Table 5 indicates a significant decrease in cefoxitin MICs up to 0.038 mM of PAßN for CLX concentrations 0.094 and 0.117 mM. This PAßN concentration threshold was also observed for increasing the nalidixic acid activity on the same strains (data not shown).

**Table 5:** PAßN dose-effect on MICs of cefoxitin tested alone and with two different sub-inhibitory concentrations of cloxacillin towards 4 representative *K. pneumoniae* strains.

	MIC (mg/L) of FOX													
Strains	Alone	+CLX*	+0.094 mM CLX+PAßN [mM (mg/L)]						+0.117 mM CLX+PAßN [mM (mg/L)]					
			0.009 (5)	0.019 (10)	0.038 (20)	0.058 (30)	0.077 (40)	0.096 (50)	0.009 (5)	0.019 (10)	0.038 (20)	0.058 (30)	0.077 (40)	0.096 (50)
ATCC 11296	8	8	8	8	4	1	1	1	8	8	2	0.5	0.25	0.25
KPBj 6	64	64	64	32	16	16	8	8	32	32	16	8	8	8
KPBj 7	64	64	64	32	8	2	2	2	32	32	4	1	0.5	0.5
KPBj 9	128	64	128	64	16	8	4	4	64	64	4	2	1	1

*, same results were obtained in the presence of 0.094 mM or 0.117 mM of CLX.

FOX: cefoxitin, CLX: cloxacillin.

To evaluate the synergic effect conferred by cloxacillin and PAßN, with regard to cefoxitin activity, differentß-lactam molecules were tested in the same conditions (Table 6). A synergic effect was noted with amoxicillin, piperacillin, cefepime, ceftazidime and ertapenem indicating that the antibiotic activity of these ß-lactams was increased in the presence of PAßN plus cloxacillin. As for nalidixic acid, no MIC change was observed with chloramphenicol, ofloxacin and erythromycin when cloxacillin was added to PAßN (data not shown). Finally, addition of clavulanic acid to cefoxitin+PAßN with or without cloxacillin did not modify cefoxitin MICs. On the contrary, addition of clavulanic acid to the amoxicillin, PAßN and cloxacillin mixture led to a higher decrease in amoxicillin MICs (Table 7).

**Table 6:** MICs of β-lactam molecules tested alone and with fixed concentrations of PAβN and cloxacillin towards 4 representative *K. pneumoniae* strains.

Strain	Mol. added	MIC mg/L														
		AMX			PIP			FEP			CAZ			ERT		
	PAβN*	−	+	+	−	+	+	−	+	+	−	+	+	−	+	+
	CLX^♦^	−	−	+	−	−	+	−	−	+	−	−	+	−	−	+
ATCC 11296		256	128	16	32	32	0.125	0.125	0.125	0.0015	1	0.25	0.125	0.015	0.015	0.007
KBPj 6		1024	1024	256	64	64	2	0.5	0.5	0.125	0.5	0.5	0.125	0.25	0.25	0.06
KPBj 7		512	512	32	64	64	0.25	0.5	0.5	0.06	1	0.5	0.06	0.06	0.06	0.007
KPBj 9		2048	2048	64	128	128	0.5	1	0.5	0.03	1	0.5	0.125	0.25	0.25	0.03

Mol: molecule, *: 0.096 mM, ^♦^: 0.117 mM, CLX: cloxacillin, AMX: amoxicillin, PIP: piperacillin, FEP: cefepime, CAZ: ceftazidime, ERT: ertapenem.

**Table 7:** MICs of cefoxitin and amoxicillin tested alone and with PAβN, cloxacillin and clavulanic acid towards 4 representative *K. pneumoniae* strains.

Strain	Mol. added	MIC mg/L												
		FOX							AMX					
	PAβN*	−	+	+	+	+	+	+	−	+	−	+	+	+
	CLX^♦^	−	−	+	−	−	+	+	−	−	−	−	+	+
	CLA 2^Δ^	−	−	−	+	−	+	−	−	−	+	+	−	+
	CLA 20^†^	−	−	−	−	+	−	+	−	−	−	−	−	−
ATCC 11296		8	8	0.25	8	8	0.5	0.5	256	128	2	2	16	0.5
KPBj 6		64	64	8	32	16	16	8	1024	1024	4	4	256	8
KPBj 7		64	32	0.5	32	16	1	1	512	512	8	8	32	4
KPBj 9		128	64	1	64	64	2	1	2048	2048	4	4	64	4

Mol: molecule, FOX: cefoxitin, AMX: amoxicillin, CLX: cloxacillin, CLA: clavulanic acid, *: 0.096 mM, ^♦^: 0.117 mM, ^Δ^: 2 mg/L, ^†^: 20 mg/L.

## Discussion

Various mechanisms of β-lactam resistance have been described in *K. pneumoniae*. The most common mechanism consists of various plasmid-mediated -β-lactamases, the most recently described being the carbapenemases KPC [17]. Reduced outer membrane permeability has also been described and generally in association with plasmid-mediated β-lactamases [4]. If the involvement of efflux activity has been clearly demonstrated in *K. pneumoniae* clinical isolates with regard to various antibiotic families, including chloramphenicol, tetracyclines and quinolones, it has never been the case with respect to β-lactam molecules [12]. We hypothesized that such a mechanism could be active in a group of *K. pneumoniae* clinical isolates representing approximately 5% of *K. pneumoniae* isolates in our hospital over the last ten years. This expectation was based on a paradoxal β-lactam resistant phenotype (reduced susceptibility to cefoxitin and susceptibility to both amoxicillin+clavulanic acid and extended spectrum cephalosporins) associated with a conjoint chloramphenicol and quinolone resistance.

Therefore, expression of AcrAB-TolC, two components of the major pump in the *Enterobacteriaceae* [9], [10] was studied in eleven *K. pneumoniae* isolates in comparison with strain ATCC 11296. These eleven isolates were clonally unrelated strains, belonged to the different *bla* gene-based groups of *K. pneumoniae* and were free of plasmid-mediated AmpC enzymes [8]. Some isolates produced a higher AcrAB-TolC signal compared to strain ATCC 11296 as previously reported in a collection of Turkish multi-drug resistant isolates [12]. In addition, we found that the production of porins, immunorelated to OmpC and OmpF porins, was quite similar to that of strain ATCC 11296 except for one strain, KPBj6, which exhibited a porin deficient phenotype. The OmpA level indicated that this strain exhibited a porin deficient profile without a pleiotropic alteration of membrane proteins [18]. By using PAßN, a well-known EPI [14], [15], we noted that the chloramphenicol, nalidixic acid, ofloxacin, erythromycin and cloxacillin MICs decreased substantially. These antibiotics are well-known substrates of efflux pumps but this is the first sound evidence that cloxacillin is an efflux pump substrate in *K. pneumoniae* isolates [19]. The addition of sub-inhibitory concentrations of cloxacillin to PAßN induced a significant restoration of ß-lactam activities in the pioneer competitive assay developed here. This effect was observed with the different ß-lactam molecules tested especially with cefoxitin, amoxicillin, piperacillin and cefepime. No MIC decrease was observed with the other antibiotic families tested in the same experimental conditions.

Interestingly, the 16-fold decrease in amoxicillin MIC obtained in the presence of both cloxacilin and PAβN was doubled when clavulanic acid which inhibits hydrolytic activity of the *K. pneumoniae* chromosomal β-lactamase was added (Table 7). As no effect of clavulanic acid was detected when it was added to the association cefoxitin/PAβN, this indicates that clavulanic acid is not a pump substrate or presents a weak affinity for efflux pump sites compared to cloxacillin added to the same association (cefoxitin/PAßN). Therefore, all these findings strongly suggest that the innate amoxicillin resistance observed in *K. pneumoniae* would be due to amoxicillin efflux for a part and amoxicillin hydrolysis for the other part. Another balance seems to exist with tazobactam, which is another inhibitor of class A-enzyme. Indeed, adding PAßN to the association piperacillin/tazobactam resulted in a significant decrease in MIC of this association while no increase in susceptibility was noted when PAßN was added to piperacillin alone. This finding strongly suggests that tazobactam is an efflux substrate that exhibitss a reduced affinity for the pump sites compared to cloxacillin. Interestingly, the study of ligand-transporter interaction carried out with purified AcrB has reported the presence of affinity sites exhibiting different affinity constants (KD values from 5.5 to 74.1 µM) for various substrates [20].

The restoration of ß-lactam activity observed in the presence of both EPI and cloxacillin showed that cloxacillin may act as a direct competitor for ß-lactam efflux. This competitive effect was obtained at low concentration of cloxacillin compared to the cloxacillin MIC found for the *K. pneumoniae* isolates and with the low amounts of PAßN classically used to inhibit the efflux of chloramphenicol and quinolones [12]. This increase in ß-lactam susceptibility, described even in ATCC strain, demonstrates the existence of a ß-lactam efflux pump expressed at low level in *K. pneumoniae*. Interestingly, we observed a noticeable PAßN-susceptible efflux of cloxacillin in strains KPBj 1 and ATCC strain in contrast to the PAßN-susceptible efflux of chloramphenicol. In addition, a PAßN-susceptible erythromycin efflux was also noted and we have previously demonstrated that macrolide efflux should be independent to AcrAB system [21]. Therefore, as the susceptibility increase does not necessarily require an AcrAB-TolC over expression, two hypotheses may be proposed to explain our findings:

1- The level of AcrAB-TolC expressed in the *K. pneumoniae* isolates is sufficient to maintain the ß-lactam intracellular concentration under the threshold required for antibacterial activity. If it was the case, due to the presence of different affinity sites located inside the AcrB pump, as mentioned during previous co-crystalization analyses and as suggested by the recent proposed model, it would be possible that the gain generated by conjoint addition of EPI and cloxacillin be due to a synergistic effect [22], [23]. In the same time, EPI would induce a steric hindrance inside the general cavity and cloxacillin would generate a competitive occupation of a ß-lactam affinity site [22]. This attractive hypothesis fits well with the AcrB vestibule entry located at the periplasmic surface of inner membrane, the report mentioning the respective location of PAßN and other ligands inside AcrB cavities, and with the idea of a flux competition conferred by a saturation of pump channel associated with occupation of specific sites exhibiting high affinity for specific drugs (ß-lactam such as cloxacillin) which cannot modify the susceptibility of other antibiotic classes (*e.g.* quinolone, macrolide) [22]. Recent competition experiments performed with the purified AcrB pump and various ligands have demonstrated that erythromycin is not able to inhibit the binding of other molecules suggesting that different families of drugs recognize different non-overlapping sites [20]. Moreover, Elkins and Mullis have demonstrated the presence of substrate hierarchy or selectivity during competitive experiences using macrolide or steroid uptake, competition occurring inside a same structural class in *E. coli* [24].

Regarding this hypothesis it is worthwhile of note that the binding of substrates to affinity sites located inside AcrB in *Haemophilus influenzae* induces noticeable conformational change altering the reactivity of specific residues [25]. In addition, the functional rotation mechanism evidenced for the multidrug efflux pump AcrB may also support this first hypothesis by the existence of three independent efflux sites acting step by step and capable to accept different substrates due to the site flexibility during the loose state [23].

**Figure 4:**
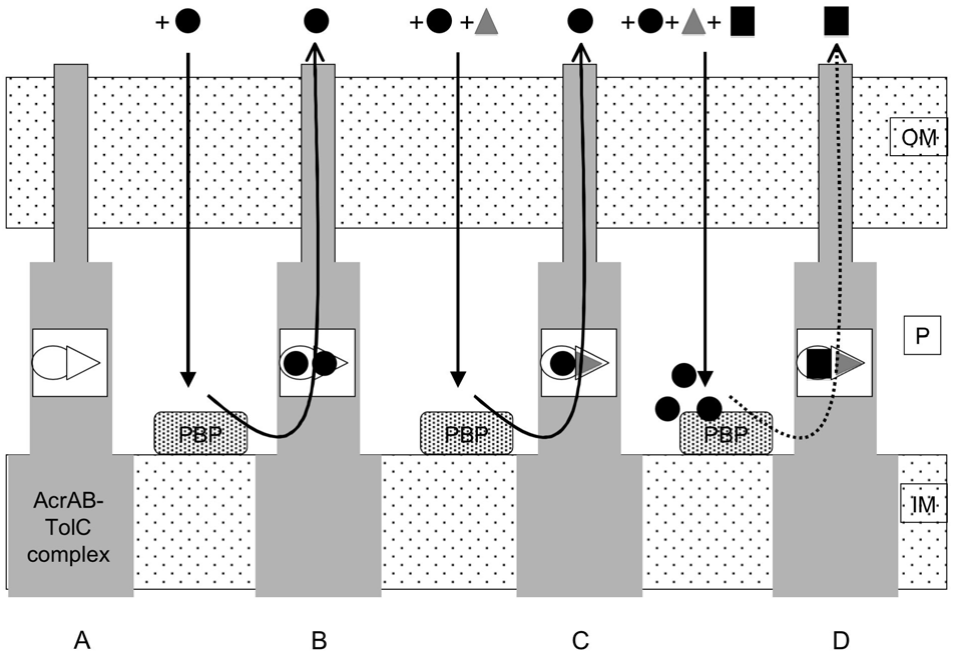
Scheme of the selective efflux of ß-lactam molecules and the effect of various molecules. A: the AcrAB-TolC efflux pump. B: the FOX efflux. C: the effect of PAßN on FOX efflux. D: the effect of PAßN+CLX on FOX efflux. OM, outer membrane; P, periplasmic space; IM, inner membrane; PBP, penicillin binding protein (ß-lactam target). Cloxacillin (CLX) and cefoxitin (FOX) are represented by black squares or black circles respectively; and grey triangles represent the PAßN. Black straight arrows represent the drug penetration through the outer membrane and the black curved lines represent the drug efflux through the efflux pumps. Bold and dotted curved lines indicated the high and low level of FOX efflux respectively. Empty circles and triangles represent the ß-lactam selective sites and the non-selective sites, respectively. PAßN is able to bind to the non-selective site located inside the pump cavity and at a lesser extent (due to affinity) to the ß-lactam site. In the presence of ß-lactam (CLX or FOX), the ß-lactam site is preferentially occupied by the ß-lactam molecules. For clarity reasons, only the first hypothesis was presented. For the second hypothesis (two pumps), the different drug affinity sites (non-selective and ß-lactam selective sites) may be distributed in the two efflux pumps acting in the same time in resistant strains.

2- The second hypothesis is that there are two pumps acting in these isolates or that selected mutations, in the efflux pump, may increase the selectivity for some substrates over other transported molecules in clinical isolates. In recent reports, several teams have mentioned the activity of pumps, different from AcrAB, which would be involved in antibiotic expel [for a review see 10], [19]. In this case, the respective affinity for the various substrates, ß-lactams, quinolones and macrolides, could be different as previously observed with the Mex pump family in *P. aeruginosa* [26]. Moreover, this difference also would concern PAßN for which some variations have been reported concerning the range of its efflux inhibitory spectrum [14], [27]. In this condition, two pumps or a selective-modified one would be functioning. In the first case, one expels quinolone, macrolides, PAßN, and also ß-lactams but with a lower efficiency/selectivity, the other having a high affinity for ß-lactam and a reduced affinity for PAßN. In the second case, the affinity for specific antibiotics being increased in the modified pump, the efflux blockage needs a competitive substrate that belongs to the same antibiotic structural class to be effective. This may reflect the variation in the immunodetected signal of efflux pump components obtained with our antibodies. As mentioned in the two hypotheses, the presence of PAßN is necessary in addition to the efficient competitor (CLX). This may support the presence of separate and discrete pockets inside the efflux channel which exhibit different affinity constants and which recognize various molecules (*e.g.* PAßN and CLX). In the absence of CLX, the PAßN affinity is not sufficient to impair FOX recognition, and in the absence of PAßN the corresponding non-specific site may accommodate FOX molecules with a reduced but significant efficiency to expel the drug (Figure 4).

Whatever the number and the selectivity of pump acting in these isolates, it is clearly demonstrated that efflux plays a key role in the ß-lactam uptake and susceptibility in *K. pneumoniae*. It is also clear that this role has been largely underestimated if we take into account the involvement of efflux in the acquisition of additional mechanisms of resistance as shown in *Salmonella* sp. and *Campylobacter* sp. [28], [29]. In addition, this study may explain previous reports describing a ß-lactam resistance level without consistent description of enzyme production or porin alteration and evoking a possible role of efflux in *K. pneumonia* [7]. From the pioneer studies of H. Nikaido on the *in vitro* efflux pumps activities and the model of efflux mechanism as vacuuming the periplasm, this study clearly highlights the clinical impact of efflux activity in ß-lactam susceptibility for the first time in *Enterobacteriaceae* [10], [30], [31]. This result is especially important with regard to the previous observations showing that some ß-lactams stimulate the expression of efflux pumps in infecting enterobacteria [32]. *In vitro* experiments have documented the role of *mar* cascade in the selection of resistant strains expressing efflux pumps under ß-lactam stress [32]. The emergence of *K. pneumoniae* isolates exhibiting a ß-lactam efflux, associated or not with a failure of porin expression, directly involved in antibiotic resistance is a predicted event. This may occur in different types of infections and we described here this bacterial strategy which concerns a number of isolates collected during a recent period and which exhibited a specific antibiotic resistance pattern.

This is the first time that the efflux involvement is clearly demonstrated to impact on clinical ß-lactam susceptibility in *Enterobacteriaceae*. This effect observed in our isolates is especially worrying due to the multiple factors (genetic and chemical) that activate the expression of efflux mechanisms in bacteria belonging to natural flora or possibly involved in nosocomial pathogens.

## Materials and Methods

### Bacterial strains and clinical data

Eleven *K. pneumoniae* isolates collected over a period of 5 years (2000–2005) in Beaujon hospital were studied. Ten of them had been isolated from blood and one from drainage fluid (Table 1). The majority of the patients infected by these isolates had hepatogastroenterological diseases (9/11) and 6 were hospitalized in ICU when the *K. pneumoniae* infection occurred. *K. pneumoniae* strains ATCC 11296 and 138831 were used as reference strains.

### Molecular epidemiology typing

The eleven isolates were typed by Random Amplified Polymorphism DNA (RAPD) [33].

### ß-lactamase determination

*bla* genes encoding AmpC and TEM ß-lactamases were identified by PCR [34]. The chromosomal *bla* gene of the eleven isolates were amplified and then sequenced as described [8].

### Immunocharacterisation of membrane transporters

Exponential-phase bacteria in LB broth were pelleted and solubilized in boiling buffer at 96°C [12]. Total cell protein (OD600 = 0.01 corresponding to equal protein per well) was loaded onto a SDS-polyacrylamide gel (10% polyacrylamide, 0.1% SDS) [12], [18], [35]. Proteins were electro-transferred onto nitrocellulose membranes in transfer buffer. An initial saturating step was performed overnight at 4°C with Tris-buffered sodium (TBS: 50 mM Tris-HCl pH 8.0, 150 mM NaCl) containing skimmed milk powder (10%). The nitrocellulose sheets were then incubated in TBS containing skimmed milk powder (5%) and Triton X-100 (0.2%) for 2 h at room temperature in the presence of polyclonal antibodies (1∶2,000 dilution) directed against denatured OmpF porin, denatured OmpC porin, OmpA [18] or against denatured AcrA or TolC [12], [16]. It is important to mention that our previous studies have demonstrated a strong cross-immunoreactivity between *E. coli* and *K. pneumoniae* porins [12], [13] due to the conservations of common antigenic regions [35]. Polyclonal antibodies directed against *E. coli* OmpC and OmpF porins have been used for the detection of expressed porins in the various *K. pneumoniae* strains. The detection of antigen-antibody complexes was performed with alkaline phosphatase conjugated AffinitiPure goat anti-rabbit IgG antibodies [18].

### Antibiotic susceptibility tests

Bacteria were grown in Luria–Bertani (LB) or Mueller–Hinton (MH) broth at 37 C. Susceptibility to amoxicillin (Sigma®), amoxicillin+clavulanic acid (Glaxo SmithKline) piperacillin (Sanofi Aventis®), piperacillin+tazobactam (Wyeth®), cefoxitin (Sigma®), ceftazidime (Sigma®), cefepime (Sigma®), ertapenem (MSD®), cloxacillin (Astellas Pharma), chloramphenicol (Sigma®), nalidixic acid (Sigma®), ofloxacin (Sigma®), and erythromycin (Fluka Biochemika®) was determined by broth dilution method, as previously described [18], [36]. Minimal inhibitory concentrations (MICs) were determined with an inoculum of 10^6^ CFU in 1 mL of MH broth containing two-fold serial dilutions of each antibiotic. Isolates were classified as susceptible, intermediately susceptible or resistant to the antibiotics tested according to the Antibiogram Committee of the French Society for Microbiology (http://www.sfm.asso.fr).

The efflux pump inhibitor (EPI) phenylalanine arginine ß-naphthylamide (PAßN) was used as previously reported: MICs of each antibiotic were determined in the presence of PAßN [36]. To evaluate a possible role of efflux in the ß-lactam activity, we developed an *in vitro* assay using different conditions: ß-lactam, ß-lactam+EPI, ß-lactam+EPI+sub-inhibitory concentration of cloxacillin, ß-lactam+EPI+clavulanic acid, and ß-lactam+EPI+sub-inhibitory concentration of cloxacillin+clavulanic acid on four *K. pneumoniae* strains (ATCC 11296, KPBj 6, KPBj 7 and KPBj 9). Nalidixic acid belonging to another structural antibiotic family was used as internal standard. The results were scored after 18 h at 37°C and were expressed as MICs. The MICs for PAßN were 256–512 mg/L in the various *K. pneumoniae* isolates.

### Plasmid- and GyrA/ParC-mediated resistance to quinolones

Plasmid- and chromosome-encoded quinolone resistance determinants (*qnr A, B* and *S*, *gyrA* and *parC* genes) were studied [37], [38].
